# A hybrid Anyon-otto thermal machine

**DOI:** 10.1038/s41534-026-01328-6

**Published:** 2026-07-24

**Authors:** Mohit Lal Bera, Joyce Kwan, Armando Pérez, Miguel A. García-March, Ravindra Chhajlany, Tobias Grass, Maciej Lewenstein, Utso Bhattacharya, Sourav Bhattacharjee

**Affiliations:** 1 Departamento de Física Teórica and IFIC, Universitat de València-CSIC, Burjassot (València), Spain; 2 ICFO-Institut de Ciéncies Fotóniques, The Barcelona Institute of Science and Technology, Av. Carl Friedrich Gauss 3, Castelldefels (Barcelona), Spain; 3 JILA, NIST, and Department of Physics, University of Colorado, Boulder, CO USA; 4 IUMPA - Instituto Universitario de Matemática Pura y Aplicada, Universitat Politècnica de València, València, Spain; 5 Institute of Spintronics and Quantum Information, Faculty of Physics and Astronomy, Adam Mickiewicz University, Poznan, Poland; 6 DIPC - Donostia International Physics Center, Paseo Manuel de Lardizábal 4, San Sebastián, Spain; 7 IKERBASQUE, Basque Foundation for Science, Plaza Euskadi 5, Bilbao, Spain; 8 ICREA, Pg. Lluis Companys 23, Barcelona, Spain; 9 Institute for Theoretical Physics, ETH Zurich, Zurich, Switzerland; 10 Max Planck Institute for the Physics of Complex Systems, Nöthnitzer Str. 38, Dresden, Germany

**Keywords:** Materials science, Physics

## Abstract

We propose a four-stroke quantum thermal machine based on the 1D anyon Hubbard model, which is capable of extracting the excess energy arising from anyon exclusion statistics at low temperature into finite work. Defining a hybrid anyon-Otto (HAO) cycle, we find that the low-temperature work, in the absence of any interactions, is maximized in the pseudo-fermionic limit, where the anyons most closely resemble free fermions. However, when weak interactions are introduced, the work output is no longer maximized at the bosonic or pseudo-fermionic extremes but instead peaks at intermediate statistical angles. This clearly demonstrates that interactions and anyonic statistics conspire non-trivially to enhance performance, with interacting anyons offering greater quantum thermodynamic advantage than either bosons or pseudo-fermions, in this regime. Furthermore, we also outline an experimental protocol to realize the HAO cycle using ultracold atoms in an optical lattice.

## Introduction

In recent times, a plethora of models of quantum thermal machines (QTMs) have been proposed and analyzed to understand the emergence of thermodynamic principles from quantum dynamics^1,2^. The existence, albeit mostly theoretical, of such a large number of models can be attributed to two reasons. Firstly, the nondeterministic and destructive nature of generic quantum measurements makes it difficult to uniquely and unambiguously define the quantum equivalents of classical work and heat. Secondly, genuine quantum phenomena have been shown to alter the performance of QTMs in a myriad of ways, depending on how they are incorporated into the model, for example, in the form of coherence in the system^3–5^, engineered heat baths^6–13^, many-body effects^14–22^, etc. In addition to theoretical investigations, progress has also been made in the experimental realization of QTMs^23–27^.

The simplest and most commonly studied models of QTMs are those based on the four-stroke quantum Otto cycle^2^. The standard Otto cycle consists of two work strokes and two thermalization strokes. In the former, a system is made to evolve unitarily through a quench in some parameter of the Hamiltonian, with the energy change of the system associated with work. The work strokes are interceded by the thermalization strokes in which the system evolves dissipatively in contact with a heat bath, the energy change being associated with the heat transferred. At the end of the full cycle, the system returns to its initial state with a net conversion of quantum heat into quantum work or vice versa. The direction of heat flow and work output determines the operating mode of the cycle, with the thermodynamically allowed (respecting the second law) modes being the engine, refrigerator, accelerator, and heater. The engine (refrigerator) mode is characterized by a net heat transfer from the hotter (colder) to the colder (hotter) bath, with a net work output (input). In accelerator mode, input work is used to boost the heat transfer from the hotter to the colder bath, while in heater mode, input work is fully converted into heat and dumped into each of the baths.

The Otto cycle operating in the engine mode, by definition, relies on thermal energy for work output; the latter thus vanishes in the limit of zero temperature of the heat baths. However, quantum statistical properties depend crucially on the nature of particles at low temperatures, and this has recently been exploited to design and experimentally realize the Pauli engine^25^. In this variant of the regular Otto engine, the thermalization strokes are replaced by the so-called Pauli strokes, in which the system undergoes a bosonization or fermionization process. Specifically, rather than thermalizing with a heat bath, the statistical nature of the system is altered by tuning it from a molecular Bose condensate to a Fermi gas or vice versa. By virtue of the Pauli exclusion principle, the system has a higher energy ground state in the fermionic state as compared to the bosonic state. The Pauli engine operates by extracting this difference in ground state energy, defined as the Pauli energy, into useful work. Importantly, the Pauli engine has no classical analog and is purely driven by quantum statistical phenomena. To this end, we note that the relation between the performance of QTMs and the statistical properties of the system has been explored in a number of other works, especially with respect to fermionic and bosonic statistics^28–34^.

**Fig. 1:**
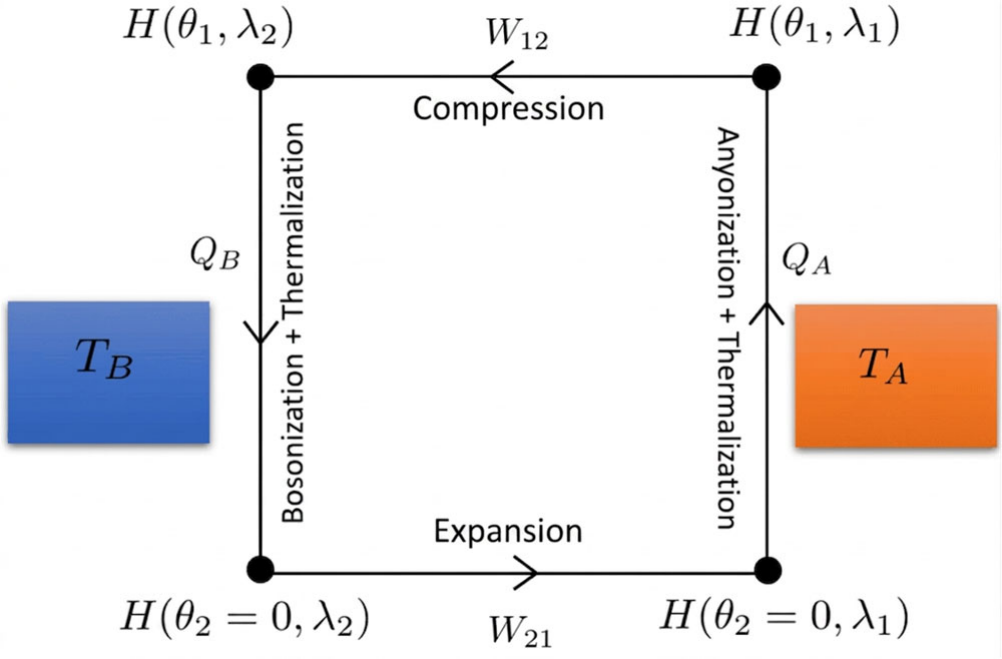
Schematic representation of the HAO cycle. The two unitary work strokes— expansion and compression, are implemented through an explicit change of the Hamiltonian parameter *λ* ∈ {*J*, *U*}. The heat strokes consist of thermalization with one of the heat baths with temperature *T*_*A*_ or *T*_*B*_ and a change in the statistical parameter *θ*.

In parallel, it has also been shown in recent times that exotic quantum particles such as anyons^35–42^, including impurity excitations in fractional quantum Hall liquids which exhibit fractional angular momentum and effective anyonic statistics^43,44^, and paraparticles^45,46^, satisfy statistical properties different from bosons or fermions. The performance of QTMs based on systems satisfying such non-trivial statistics has largely remained unexplored, except for recent models based on few-body anyonic systems^47–50^. In this work, we establish how anyonic statistics lead to non-trivial performance enhancement in a many-body QTM, which, importantly, can be realized using current experimental platforms. Specifically, we define and analyze a hybrid anyon-Otto (HAO) cycle (see Fig. 1), based on the anyon Hubbard model (AHM)^51–65^, in which the statistical properties of the particles can be continuously tuned from boson-like to pseudo-fermion-like (satisfying anti-commutation relations only when particles are on different sites). We contrast the operation of the HAO cycle with the Otto cycle in the same temperature and parameter regime, except that the statistical properties of the system remain unchanged throughout the Otto cycle. We find that the HAO cycle can produce a finite work output even in the limit of vanishing temperatures of the baths, due to the presence of an anyon energy, defined analogously to the Pauli energy. In the absence of explicit interactions between the anyons, we demonstrate that the HAO cycle can operate in an inverse accelerator mode for finite but small temperatures. This mode, characterized by a heat transfer from colder to hotter bath and a net work output, exhibits an apparent violation of the second law, which is reconciled when the work production in the anyonization stroke is appropriately accounted for. In the presence of weak interactions between the anyons, we observe that anyonic statistics boosts the performance of the QTM at low temperatures, manifested in the form of higher work output as compared to bosonic or pseudo-fermionic statistics. Finally, we propose how the HAO cycle can be realized experimentally through a feasible extension of the experimental protocol used for the recent realization of the AHM^51,61,62^, thus rendering the possibility of direct experimental verification of our results.

## Results

### Anyon Hubbard model

The 1D AHM is realized by introducing a synthetic gauge field in the hopping term of the Bose Hubbard model^53^,

1 $$H=-J\mathop{\sum }\limits_{j=1}^{L}\left({\hat{b}}_{j}^{\dagger }{e}^{-i{\hat{n}}_{j}\theta }{\hat{b}}_{j+1}+h.c.\right)+\frac{U}{2}\mathop{\sum }\limits_{j=1}^{L}{\hat{n}}_{j}\left({\hat{n}}_{j}-1\right),$$

where *L* is the total number of sites, ${\hat{b}}_{j}$ are the bosonic annihilation operators, ${\hat{n}}_{j}={\hat{b}}_{j}^{\dagger }{\hat{b}}_{j}$ is the number operator, *J* is the tunneling amplitude, *U* is the interaction strength, and *θ* is a phase parameter. Defining the annihilation operators ${\hat{a}}_{j}={e}^{-i\theta {\sum }_{1\le l\le j-1}{\hat{n}}_{j}}{\hat{b}}_{j}$, the equivalent anyon model is obtained,

2 $$H=-J\mathop{\sum }\limits_{j=1}^{L}\left({\hat{a}}_{j}^{\dagger }{\hat{a}}_{j+1}+h.c.\right)+\frac{U}{2}\mathop{\sum }\limits_{j=1}^{L}{\hat{n}}_{j}\left({\hat{n}}_{j}-1\right),$$

where ${\hat{n}}_{j}={\hat{b}}_{j}^{\dagger }{\hat{b}}_{j}={\hat{a}}_{j}^{\dagger }{\hat{a}}_{j}$. The operators ${\hat{a}}_{j}$ satisfy the anyonic commutation relations, ${\hat{a}}_{j}{\hat{a}}_{k}^{\dagger }-{e}^{i\theta {\rm{sgn}}(j-k)}{\hat{a}}_{k}^{\dagger }{\hat{a}}_{j}={\delta }_{jk}$ and ${\hat{a}}_{j}{\hat{a}}_{k}-{e}^{i\theta {\rm{sgn}}(j-k)}{\hat{a}}_{k}{\hat{a}}_{j}=0$. The phase parameter *θ* thus provides a direct control over the statistical properties of the particles. It is straightforward to see that the bosonic statistics are recovered in the limit *θ* → 0. In contrast, *θ* → *π* leads to pseudo-fermionic statistics as the anyon operators anti-commute on different lattice sites but commute on the same site.

### Hybrid anyon-Otto cycle

The HAO cycle that we propose here is a four-stroke cycle that inherits operational characteristics from both the Pauli and Otto cycles. Thus, a hallmark of this cycle is its ability to function both as an anyon engine (defined analogously to the Pauli engine) at low temperature and as the standard Otto engine at high temperature. Before defining the HAO cycle, we recall that in the Otto cycle, work and heat are commonly associated with the energy exchange in strokes undergoing exclusively unitary or dissipative dynamics, respectively,

3 $${W}_{otto}=\int{\rm{Tr}}\left[\rho \frac{\partial H}{\partial t}\right]dt,$$

4 $${Q}_{otto}=\int{\rm{Tr}}\left[\frac{\partial \rho }{\partial t}H\right]dt,$$

where *ρ* is the density operator representing the state of the system. In other words, a unitary evolution with a time-dependent Hamiltonian only contributes to the work, while a dissipative evolution, such as thermalization with a heat bath, only results in heat transfer in the Otto cycle.

We now define the HAO cycle as follows. At the start of the cycle, we assume that the system with Hamiltonian *H*(*θ*_1_, *λ*_1_) is in equilibrium with a thermal bath with temperature *T*_*A*_ (inverse temperature *β*_*A*_), and *λ* represents either of the parameters *J* or *U* appearing in the Hamiltonian. The initial state is therefore a thermal Gibbs state ${\rho }_{1}\equiv {e}^{-{\beta }_{A}H({\theta }_{1},{\lambda }_{1})}/{\rm{Tr}}\left[{e}^{-{\beta }_{A}H({\theta }_{1},{\lambda }_{1})}\right]$. The HAO cycle then consists of the following sequential strokes (see Fig. 1):

1. Unitary compression: (λ_1_ → λ_2_, isolated)— The Hamiltonian parameter is ramped from *λ*_1_ to *λ*_2_ over a time *τ*. The system evolves unitarily to a state ${\rho }_{2}={U}_{12}^{\dagger }(\tau ){\rho }_{1}{U}_{12}(\tau )$ during this time interval. The change in the energy expectation value is thus associated with work performed,

5 $${W}_{12}=-\left[{\rm{Tr}}\left({\rho }_{2}H({\theta }_{1},{\lambda }_{2})\right)-{\rm{Tr}}\left({\rho }_{1}H({\theta }_{1},{\lambda }_{1})\right)\right].$$

2. Thermalization *B*: (*θ*_1_ → *θ*_2_, contact with heat bath)—This stroke consists of two sub-strokes—the phase parameter is tuned from *θ*_1_ to *θ*_2_ followed by thermalization with a heat bath of temperature *T*_*B*_. Note that the two sub-strokes can be carried out simultaneously, provided that the phase parameter is changed over a time interval much shorter than the thermalization timescale. The steady state reached is thus given by, ${\rho }_{3}={e}^{-{\beta }_{B}H({\theta }_{2},{\lambda }_{2})}/{\rm{Tr}}\left[{e}^{-{\beta }_{B}H({\theta }_{2},{\lambda }_{2})}\right]$. The change in the energy expectation value in this stroke has two contributions, one from the energy dissipated to the heat bath and the other from the work done in changing the phase parameter. However, for reasons that we shall clarify below, we associate the total energy change in this stroke with heat transfer,

6 $${Q}_{B}=\left[{\rm{Tr}}\left({\rho }_{3}H({\theta }_{2},{\lambda }_{2})\right)-{\rm{Tr}}\left({\rho }_{2}H({\theta }_{1},{\lambda }_{2})\right)\right].$$

3. Unitary expansion: (λ_2_ → λ_1_, isolated)—As in the case of the unitary compression stroke, the system evolves unitarily to ${\rho }_{4}={U}_{21}^{\dagger }(\tau ){\rho }_{3}{U}_{21}(\tau )$ and the change in the energy expectation value corresponds to work performed *W*_21_,

7 $${W}_{21}=-\left[{\rm{Tr}}\left({\rho }_{4}H({\theta }_{2},{\lambda }_{1})\right)-{\rm{Tr}}\left({\rho }_{3}H({\theta }_{2},{\lambda }_{2})\right)\right].$$

4. Thermalization A: (*θ*_2_ → *θ*_1_, contact with heat bath)—In the final stroke, the phase parameter is restored to its initial value *θ*_1_ and the subsequent thermalization with the heat bath at temperature *T*_*A*_ results in the system returning back to its initial state *ρ*_1_. Thus,

8 $${Q}_{A}=\left[{\rm{Tr}}\left({\rho }_{1}H({\theta }_{1},{\lambda }_{1})\right)-{\rm{Tr}}\left({\rho }_{4}H({\theta }_{2},{\lambda }_{1})\right)\right].$$

The total work *W* = *W*_12_ + *W*_21_, *Q*_*A*_ and *Q*_*B*_, satisfies energy conservation (first law) *W* = *Q*_*A*_ + *Q*_*B*_. The difference between the HAO cycle defined above and the standard quantum Otto cycle lies in the two modified thermalization strokes. In the latter case, the statistical properties of the quantum model remain unchanged, and thus the heat change is solely associated with the energy gained or lost to the heat baths. In contrast, thermalization in the HAO cycle is accompanied by an explicit change of statistical properties through the parameter *θ*. It is important to note that ramping the phase parameter modifies the Hamiltonian itself and thus technically amounts to performing a certain amount of work. However, in the anyon equivalent of the model defined in Eq. (2), *θ* implicitly controls the statistical properties and hence the equilibrium configuration of the system. Since the purpose of the HAO engine is to convert both thermal energy and anyon energy into useful work, we associate the total energy change in the modified thermalization strokes only with heat energy.

### The non-interacting limit **U=0**

**Fig. 2:**
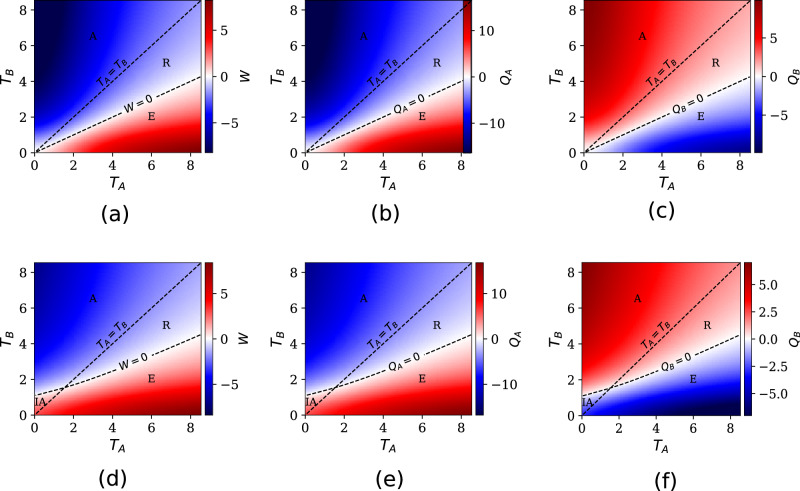
Operating modes of the HAO cycle in the non-interacting limit. Work output *W* (**a**, **d**), heat exchanged with the baths *Q*_*A*_ (**b**, **e**), and *Q*_*B*_ (**c**, **f**), in the HAO cycle as a function of the bath temperatures *T*_*A*_ and *T*_*B*_. The top panel corresponds to the standard Otto cycle with *θ*_1_ = *θ*_2_ = 0, showing the three possible working modes—engine(E), refrigerator (R), and accelerator (A). The bottom panel shows the emergence of the inverse accelerator mode (IA) in the low temperature limit for *θ*_2_ = 0, *θ*_1_ ≠ 0. The plots depicted above are obtained numerically for a chain of length *L* = 12, with number of particles *N* = 6, *U* = 0, *J*_1_ = 2.0, and *J*_2_ = 1.0.

We first consider the operation of the HAO cycle in the non-interacting limit *U* = 0 with *λ* ≡ *J*. In this limit, the Hamiltonians at different instants of time during the work strokes commute with each other, implying adiabatic evolution and thus rendering the cycle operation independent of the work stroke duration *τ*. We also assume that the total number *N* of particles remains conserved throughout the cycle. In Fig. 2a–c, we examine the possible modes of operation for the standard Otto cycle (*θ*_1_ = *θ*_2_ = 0), choosing without loss of generality *J*_1_ = 2.0, *J*_2_ = 1.0, *L* = 12, and *N* = 6. Depending on the signs of *W*, *Q*_*A*_ and *Q*_*B*_, and the relative magnitudes of *T*_*A*_ and *T*_*B*_, three modes of cycle operation can be identified. When *T*_*A*_ > *T*_*B*_, the cycle can operate either in the engine mode (E) with *Q*_*A*_, *W* > 0, *Q*_*B*_ < 0 or the refrigerator mode (R) with *Q*_*A*_, *W* < 0, *Q*_*B*_ > 0. For *T*_*A*_ < *T*_*B*_, the only possible mode of operation is the accelerator mode (A) with *Q*_*A*_, *W* < 0, *Q*_*B*_ > 0 (see Table 1). The E-R transition can be identified by the line *W* = *Q*_*A*_ = *Q*_*B*_ = 0. For *T*_*A*_, *T*_*B*_ → 0, the work output and the heat vanish identically.

**Table 1:** Summary of the different modes of operation of the HAO cycle

Mode	Total work extracted	Energy gained from hotter bath	Energy gained from colder bath
Engine (E)	+	+	−
Refrigerator(R)	−	−	+
Accelerator (A)	−	+	−
Inverse Accelerator (IA)	+	−	+
Heater (H)	−	−	−

The +(−) symbol in the second column corresponds to a net work extracted from (performed on) the system. Similarly, in the third and fourth columns, +(−) corresponds to energy gained (lost) by the system from the respective baths.

Having established the modes of operation in the Otto cycle, we now consider the situation *θ*_1_ = *π*, *θ*_2_ = 0, that is, the statistical properties of the system are altered between the pseudo-fermionic and bosonic limits during the thermalization strokes. From Fig. 2d–f, it is evident that in the large temperature limit of the baths, *T*_*A*_, *T*_*B*_ ≫ 1, the HAO cycle is identical to the Otto cycle owing to the fact that anyonic statistics can be well approximated by Boltzmann statistics in this limit. However, at low temperatures, *T*_*A*_, *T*_*B*_ → 0, the excess energy resulting from the development of anyonic exclusions begins to manifest itself in the form of the non-zero work output and heat exchanges. This leads to the emergence of an *inverse accelerator* (IA) mode—heat flows from the colder bath to hotter bath with a net work extraction, *Q*_*A*_, *W* > 0, *Q*_*B*_ < 0 for *T*_*A*_ < *T*_*B*_. As we shall show later, the IA mode does not violate the second law of thermodynamics since the anyon energy gained by the system during the anyonization process involves an additional work cost, which when included in the definition of *W* restores the second law.

**Fig. 3:**
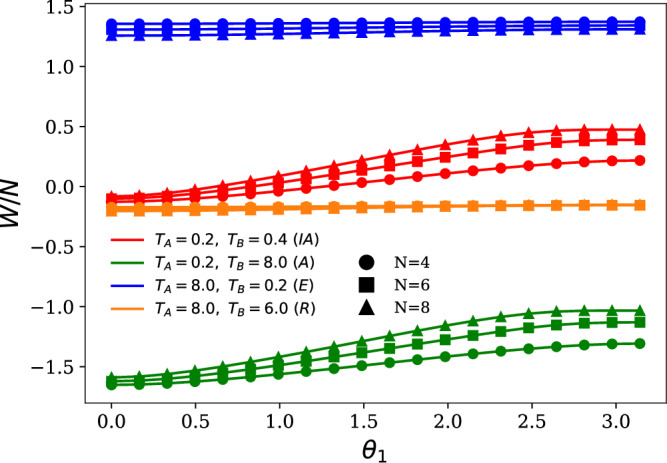
Work output in the non-interacting limit U = 0. The work output per particle in the non-interacting limit *U* = 0 is shown as a function of *θ*_1_. The anyon energy, which is built up during the anyonization stroke, is finite only when *T*_*A*_ → 0. The work output due to the anyon energy increases monotonically with *θ*_1_. The plots are obtained numerically for *L* = 12, *θ*_2_ = 0, *J*_1_ = 2.0, and *J*_2_ = 1.0.

The finite work output in the limit of vanishing temperature manifests itself not only in the pseudo-fermionic limit *θ*_1_ = *π*, but for any *θ*_1_ ≠ 0. This is demonstrated in Fig. 3, where we observe that the average work output per particle *W*/*N* increases monotonically with increasing *θ*_1_ in the inverse accelerator mode (*T*_*A*_ = 0.2, *T*_*B*_ = 0.4), provided *T*_*A*_ → 0. Similarly, the work input decreases with increasing *θ*_1_ in the accelerator mode (*T*_*A*_ = 0.2, *T*_*B*_ = 8.0) in the same limit of *T*_*A*_. Note that it is sufficient for only the *T*_*A*_ to be small as the anyonization process occurs in contact with the heat bath of temperature *T*_*A*_.

### Weak interaction **U ≪ J**

**Fig. 4:**
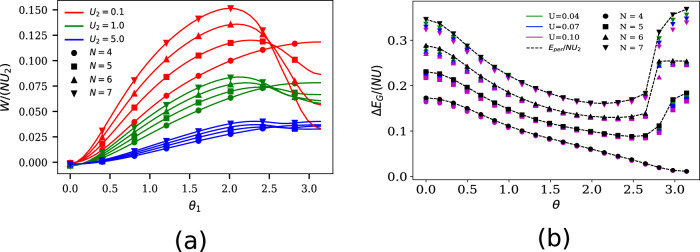
Work output in the interacting limit U≠0. **a** Low temperature (*T*_*A*_ = *T*_*B*_ = 0.1) work output per particle (scaled with *U*_2_) in the presence of finite interaction *U*_2_ ≠ 0 as a function of *θ*_1_. On approaching the weakly interacting limit *U*_2_ ≪ *J*, the work output exhibits a non-monotonic behavior with *θ*_1_ as $N\to {\frac{L}{2}}^{-}$. The plots are obtained numerically for *L* = 12, *J* = 1.0, *U*_1_ = 0, and *θ*_2_ = 0. **b** Increase in the ground state energy Δ*E*_*G*_ when interaction is switched on to a finite *U*. The increase is well approximated (black dashed lines) by a first-order correction to the ground state energy with *U* considered as a small perturbation.

We now consider the situation in which work is performed by ramping the interaction parameter *U* during the work strokes, holding *J* to a constant value. In this case, the Hamiltonian does not commute with itself at different times during the work strokes, which leads to non-adiabatic excitations for finite stroke duration *τ*. However, for simplicity, we shall assume that *τ* → *∞* as the results discussed in the following do not change qualitatively in the presence of non-adiabatic excitations. In order to ensure that the anyon energy is not affected by the interaction, we choose *U*_1_ = 0. The work output *W* at low temperature, *T*_*A*_ = *T*_*B*_ = 0.1, as a function of the statistical parameter *θ*_1_, is shown in Fig. 4a for different interaction strengths *U*_2_. In the weakly interacting limit *U* ≪ *J*, we observe that *W* is maximized for *θ*_1_ = θ^*^ such that 0 < θ^*^ < *π*, for *N*≥*L*/2. However, as the interaction strength is increased, the maxima shift towards the pseudo-fermionic limit θ^*^ = *π*. Furthermore, within the largest Hilbert-space dimensions numerically accessible to us, we do not observe any appreciable drift of θ^*^ with increasing *L* as long as the filling fraction *N*/*L* is held constant^66^. This indicates that an intermediate optimal value of *θ* is likely to persist in the thermodynamic limit.

**Fig. 5:**
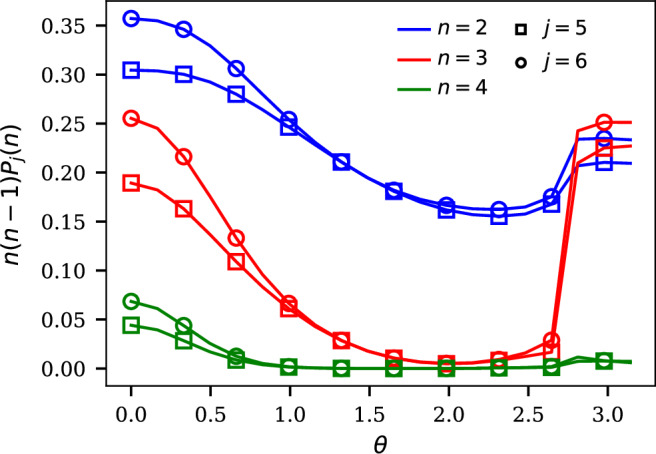
Particle occupancy probabilities in the ground state for *U* = 0. The probability *P*_*j*_(*n*) of *n* bosons (scaled with *n*(*n* − 1)) occupying the *j*th site in the chain in the ground state is shown as a function of *θ*. The plots are obtained for a system of size *L* = 12 with *N* = 6, *J* = 1.0, and *U* = 0.

To understand the above results, we first note that the dependence of *W* on *θ*_1_ arises only from the unitary compression stroke, that is, when the interaction strength is increased from *U*_1_ = 0 to *U*_2_ at a fixed *θ*_1_. In Fig. 4b, we see that the change in the ground state energy Δ*E*_*G*_ during this stroke is also minimized at intermediate values of *θ*_1_ between 0 and *π* for *U* ≪ *J* and *N*≥*L*/2. The maximization of *W* (recall that it is defined with a negative sign) observed in Fig. 4a is a direct consequence of this non-trivial behavior of the ground state energy. This can be understood as follows. Firstly, for *U* ≪ *J*, Δ*E*_*G*_ can be approximated by the first-order perturbative correction to the ground state energy of the non-interacting part of the Hamiltonian. Considering the interaction ${H}_{int}=\frac{U}{2}{\sum }_{j}{\hat{n}}_{j}({\hat{n}}_{j}-1)$ as a perturbation of magnitude *U*,

9 $$\Delta {E}_{G}\approx {E}_{per}=\langle {\psi }_{0}| {H}_{int}| {\psi }_{0}\rangle =\frac{U}{2}\mathop{\sum }\limits_{j}^{L}\left[\mathop{\sum }\limits_{n=0}^{N}n(n-1){P}_{j}(n)\right],$$

where $\left\vert {\psi }_{0}\right\rangle$ is the ground state of the system for *U* = 0, and *P*_*j*_(*n*) is the marginal probability that the *j*^*th*^ site is occupied by *n* particles in the ground state $\left\vert {\psi }_{0}\right\rangle$ (see ref. ^66^ for detail). Figure 4b shows that *E*_per_ (dashed lines) indeed approximates Δ*E*_*G*_ for *U* ≪ *J*. Secondly, from Eq. (9), it is clear that *E*_per_ depends only on the probabilities of double or higher occupancies per site. The relatively smaller magnitude of *E*_per_ for intermediate values of *θ* for *N*≥*L*/2 can therefore be attributed to smaller values of *P*_*j*_(*n* ≥ 2) at these values as compared to *θ* = 0 and *θ* = *π*. To support the above argument, we plot in Fig. 5 the marginal probability *P*_*j*_(*n*) that the *j*th bulk site (avoiding edge effects due to OBC) is occupied by *n*_*j*_ particles for a couple of neighboring bulk sites *j* = 5, 6, in the system with *L* = 12, *J* = 1.0 and *U* = 0. It can be seen from Fig. 5 that the *P*_*j*_(*n* ≥ 2) decreases initially as *θ* is increased from zero, before increasing again on approaching *θ* = *π*.

For *N* ≲ *L*/2, the monotonous reduction of the probability of high occupancy sites in the ground state as a function of *θ* is straightforward to understand. At such low fillings, the system minimizes energy by avoiding configurations that have particles on nearby sites as well as multiple particles on the same site, so as to minimize the energy cost due to the density-dependent phase associated with the hopping term in Eq. (1) for *θ* ≠ 0. However, as *N* approaches *L*/2 from below, such configurations cannot be avoided. In fact, previous results have indicated a phase transition from the superfluid phase to a partially-paired phase as *θ* → *π* for *N* ≈ *L*/2 ^53^. The partially-paired phase in particular has been shown to favor configurations with double occupancy per site, which is consistent with our observation of increasing probability of multiple particle occupations per site as *θ* → *π*. Intuitively, the origin of the above behavior can be attributed to the fact that the density-dependent phase can only acquire the values ≈±1 as *θ* → *π*, which makes higher occupation per site favorable again, unlike for intermediate values of *θ*. In ref. ^66^, we provide further numerical evidence in support of this argument.

### Reconciliation with the second law

In the previous sections, we have argued that the apparent violation of the second law of thermodynamics in the IA mode can be reconciled if the work associated with the buildup of anyon energy is accounted for. To show this explicitly, we modify the definition of work in Eq. (5) and Eq. (7) to incorporate the energy change associated with the anyonization and bosonization processes. Likewise, we subtract the same from the heat exchanges to ensure consistency with the first law. Furthermore, in this case, we assume that the anyonization/bosonization process is carried out much faster than the timescale of thermalization with the baths. This ensures that the energy change during the anyonization/bosonization process is only due to work performed by explicit change of the parameter *θ* in the Hamiltonian. Thus, the redefined work and heat are, ${\overline{W}}_{12(21)}={W}_{12(21)}-{\Delta }_{B(A)}$, $\overline{W}={\overline{W}}_{12}+{\overline{W}}_{21}$, ${\overline{Q}}_{A(B)}={Q}_{A(B)}-{\Delta }_{A(B)}$, where

10 $${\Delta }_{B(A)}={\rm{Tr}}\left[{\rho }_{2(1)}\left(H({\theta }_{2(1)},{\lambda }_{2(1)})-H({\theta }_{1(2)},{\lambda }_{2(1)})\right)\right].$$

**Fig. 6:**
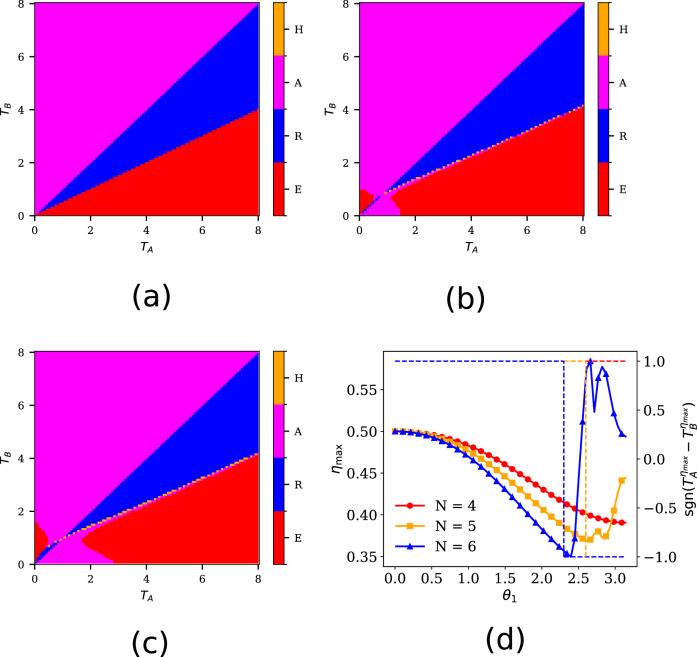
Performance of the HAO cycle corresponding to modified work $\overline{W}$ and heat $\overline{Q}$ (see Eq. (10) and discussion therein). Operation modes in the non-interacting limit *U* = 0 for *θ*_2_ = 0, and **a** *θ*_1_ = 0, **b** *θ*_1_ = 2.2, and **c** *θ*_1_ = *π*. **d** Maximum efficiency *η*_max_ calculated over 0 < *T*_*A*_, *T*_*B*_ < 8 as a function of *θ*_1_ for *θ*_2_ = 0. The dashed lines correspond to $\mathrm{sgn}({T}_{A}^{{\eta }_{\max }}-{T}_{B}^{{\eta }_{\max }})$, where ${T}_{A(B)}^{\max }$ are the temperatures of the bath for which the maximum efficiency is obtained for a fixed *θ*_1_. The numerical data are calculated for *L* = 12, *U* = 0, *J*_1_ = 2.0, and *J*_2_ = 1.0.

Since the HAO cycle operates like a regular Otto cycle in terms of $\overline{W}$ and $\overline{Q}$, it now also becomes meaningful to define the efficiency $\eta =\overline{W}/{\overline{Q}}_{A(B)}$ for *T*_*A*(*B*)_ > *T*_*B*(*A*)_, provided the cycle is operating in the engine mode. Examining the non-interacting situation with *U* = 0, *J*_1_ = 2.0, and *J*_2_ = 1.0, it is evident from Fig. 6a–c that the inverse accelerator mode does not emerge even for *θ*_1_ ≠ 0. However, the effect of changing the statistical properties with *θ*_1_ still manifests itself at low temperatures. In particular, for *θ*_1_ ≥ 2.2, the cycle is able to operate as an accelerator for a certain range of small but finite temperatures for *T*_*A*_ > *T*_*B*_. Likewise, for *T*_*A*_ < *T*_*B*_, the cycle switches from accelerator to engine mode *θ*_1_ ≥ 2.2 at small temperatures. Finally, we also note the emergence of heater mode (see Table 1) in the vicinity of the temperatures at which the direction of heat flow switches for the two baths.

It is fascinating to note that the maximum possible efficiency of the cycle when operating in engine mode is significantly enhanced for *θ*_1_ ≥ 2.2. In fact, the maximum efficiency is found to be from the engine mode at *T*_*A*_ < *T*_*B*_, which emerges for *θ*_1_ ≥ 2.2 (bottom left in Fig. 6b, c). This is illustrated in 6d, where we plot the maximum efficiency across all temperatures as a function of *θ*_1_. In the same figure, we also plot ${\rm{sgn}}({T}_{A}^{max}-{T}_{B}^{max})$ (dashed lines), where $\mathrm{sgn}({T}_{A}^{\max }-{T}_{B}^{\max })$ are the temperatures of the bath for which the maximum efficiency is obtained for a fixed *θ*_1_. For *N* = 6, we can clearly see that *η*_max_ increases drastically for *θ*_1_ > 2.5. This coincides with ${T}_{A}^{max}$ becoming smaller than ${T}_{B}^{\max }$, signifying that the higher efficiency is extracted from the newly emerged engine regime at small temperature.

### Experimental realization

The 1D AHM has already been realized experimentally using ultracold ^87^Rb atoms in an optical lattice^61^, where the tunneling amplitude is engineered to depend on the particle density in accordance with Eq. (1). In this section, we propose how the HAO thermal machine can be realized using the same experimental setup. The main challenges associated with implementing the HAO cycle on the optical lattice platform of^61^ are the following - (i) realization of a thermal bath with which the system interacts during the thermalization strokes, (ii) measurement of the energy change of the system at the end of each stroke, and (iii) requirement of higher particle density (*N* > 2) to observe the superior performance of the cycle for 0 < *θ* < *π*.

To overcome the first challenge, we propose to introduce a second set of atoms in the lattice, which acts as a bath for the atoms already present in the system. Specifically, the additional atoms that form the bath will be prepared in the superfluid state (*θ*_bath_ → 0) to facilitate a controlled tunability of the effective temperature of the bath. Similar realization of controlled bath environments has been proposed and experimentally implemented in a number of other works^67–70^, and allows for the controlled engineering of dissipation. In our setup, the bath will be located along the length *L* of the system, forming a contiguous region of the lattice adjacent to the system sites, where it is prepared in the superfluid state using the Floquet scheme described in ref. ^71^. The size of the bath will be much larger than the system, such that the system can couple to continuum modes. Independent control of the statistical parameter *θ* will be necessary for the bath and the system, as *θ*_bath_ → 0 while *θ*_system_ changes throughout the HAO cycle, and can be realized by implementing the Floquet scheme using a digital micromirror device (DMD) rather than directly modulating the intensity of the lattice. In this approach, both the system and bath are realized using the same set of modulation frequencies, with the bath corresponding to the case *θ* = 0, implemented via spatially dependent control of the relative phases of the drive. Using the DMD to modulate the intensity allows for spatial control of the statistical parameter, as the DMD can be programmed to project modulated light with different phase configurations onto contiguous regions of the lattice, thereby realizing the system and the bath, respectively. The system will be separated from the bath by an optically projected wall, implemented either by the same or another DMD, where the height of the wall is determined by the intensity of the light. By changing the intensity of the light, and therefore the height of the wall, the system can be coupled or isolated from the bath during the thermalization or work strokes, respectively. During the thermalization strokes, the height of the wall will be controlled so as to suppress leakage of particles into the bath while allowing for energy exchange between the system and the bath.

Floquet heating is an important consideration in interacting systems, as energy absorption can, in principle, lead to population of higher bands and breakdown of the single-band description. In practice, this is mitigated by choosing modulation frequencies that are off-resonant with both the low-energy scales (tunneling and interactions) and the band gap, resulting in a long-lived prethermal regime^72^. Experiments realizing the AHM have demonstrated minimal heating and good agreement with single-band predictions over timescales relevant for state preparation and observation^73^. Additional techniques, such as multi-frequency driving to suppress interband coupling, may further extend this regime^74^.

Concerning the second challenge, we shall resort to time-of-flight (TOF) measurements to find the energy *E*(*λ*, *θ*) at the end of each stroke. We illustrate the measurement protocol only for the non-interacting case (*U* = 0) here, as the same protocol will also work for weak interactions. In the absence of interactions, we recall that the work strokes consist of adiabatically tuning the parameter *J* as the system evolves unitarily, while the heat strokes correspond to a dissipative evolution of the system in contact with thermal baths, along with a simultaneous tuning of the phase parameter *θ*. The measurement protocol will therefore be as follows. Let us assume that the system is initially prepared in the state *ρ*_3_, i.e., in thermal equilibrium with the bath with temperature *T*_*B*_ and *θ* = 0. We first perform a TOF measurement to determine the energy *E*(*J*_2_, 0) of the system in this state. The system is confined by optically projected walls along both *x* and *y*, with the walls along *y* defining the length *L*, and to perform TOF, the walls along *y* are quenched to allow the system to expand within a 1D tube. Fluorescence images of the atoms are taken at successively longer times after expansion to track the expansion profile of the system, from which a velocity and therefore the energy may be extracted. Since the TOF measurement destroys the initial state, we reset the system to *ρ*_3_ before starting the HAO cycle. The compression work stroke is performed by decoupling the bath and adiabatically tuning the hopping amplitude *J*_2_ → *J*_1_ over a time interval *τ*_*W*_, during which the system evolves to *ρ*_4_. At *t* = *τ*_*W*_, the TOF measurement is performed again to find the energy *E*(*J*_1_, 0). The work *W*_21_ is therefore obtained as *W*_21_ = 〈*E*(*J*_1_, 0) − *E*(*J*_2_, 0)〉.

In the next step, we once again prepare the system in state *ρ*_3_, evolve unitarily to *ρ*_2_, followed by coupling the system to the bath with temperature *T*_*A*_. The phase parameter is then tuned to *θ* = *θ*_1_ while in contact with the bath, and we ensure that the system thermalizes with the bath for a sufficiently long time after tuning of the phase parameter is completed, before performing the TOF measurement to find *E*(*J*_1_, *θ*_1_). The heat exchanged is thus calculated as *Q*_*A*_ = 〈*E*(*J*_1_, *θ*_1_) − *E*(*J*_2_, 0)〉 − *W*_21_. Similarly, to calculate *W*_12_ and *Q*_*B*_, we shall reset the system to *ρ*_3_ in each case, and then perform TOF measurements at the end of the unitary compression and thermalization B strokes, respectively, to find *W*_12_ = 〈*E*(*J*_2_, *θ*_1_) − *E*(*J*_2_, 0)〉 − *W*_21_ − *Q*_*A*_ and ${Q}_{B}=\langle E({J}_{2},0){\prime} -E({J}_{2},0)\rangle -{W}_{21}-{Q}_{A}-{W}_{12}$, where $E({J}_{2},0){\prime}$ is the measured energy of the system after completing the cycle. We note that $E({J}_{2},0){\prime} \ne E({J}_{2},0)$ if the thermalization is not perfect in the heat exchange strokes, which will indicate an incomplete cycle.

Finally, regarding the third challenge, achieving a higher particle density (*N* > 2) is possible as long as the filling *N*/*L* ≲ 0.36^75^; otherwise, the Floquet implementation does not faithfully realize the AHM. However, we note that the underlying Floquet-engineering framework is expected to generalize to larger particle numbers through the inclusion of additional frequency components in the drive, consistent with related schemes such as that in ref. ^61^. In particular, higher driving frequencies can be used to resonantly address the interaction-energy scales associated with higher-density configurations arising from larger particle occupations. A systematic exploration of such multi-chromatic driving protocols for higher particle densities is left for future work. Furthermore, the key features of the HAO cycle, including finite work extraction at low temperatures and its dependence on the statistical parameter, do not require a high filling fraction and can be observed even for *N* ≪ *L*/2. Finally, we note that other implementations that do not involve shaking may provide a promising route toward realizing such systems at higher filling^76^.

## Discussion

In summary, we have proposed a four-stroke hybrid anyon-Otto cycle based on the 1D anyon Hubbard model which, like its Pauli counterpart, relies on exclusion statistics of anyons to derive work at low temperatures. In the absence of an explicit interaction among the anyons, we see a monotonous increase in the low-temperature work output as the statistical parameter is increased from the bosonic limit to the pseudo-fermionic limit. The presence of a finite anyon energy at low temperature leads to the emergence of an inverse accelerator mode of the cycle, which is prohibited by the second law in a regular Otto cycle. When finite but weak interactions are introduced, and at half or higher filling, the low-temperature work output no longer peaks at the bosonic or pseudo-fermionic limits. Instead, it reaches a maximum at an intermediate value of the statistical parameter, thus demonstrating that in the interacting regime, anyonic statistics can be harnessed to achieve greater work extraction than is possible with either bosonic or fermionic statistics alone. We reiterate that the inverse accelerator mode does not violate the second law in the HAO cycle; they arise from treating the anyonization stroke—which can be interpreted as a work stroke—as a “heat” source, as clarified in the final discussion. By properly incorporating the anyonic contribution and redefining the work, we show that at low temperatures the accelerator mode is replaced by an engine mode. This engine mode exhibits maximum efficiency in the anyonic limit at high *θ*, highlighting the crucial role of anyonic statistics in enhancing cycle performance. Finally, we outline an experimental protocol to verify our results using a similar experimental platform that was used to realize the AHM.

## Methods

### Numerical simulations

All the numerical simulations were performed in the bosonic representation of the AHM Hamiltonian (Eq. (1)) using the QuSpin package^77,78^. The density-dependent phase of the hopping terms cannot be directly implemented using the standard Quspin routines for constructing Hamiltonians. Hence, we explicitly encoded the hopping terms as sparse matrices and added the same to the onsite interaction terms constructed directly using QuSpin routines^79^. The full Hamiltonian was then diagonalized within Quspin for the generation of the required data.

## Supplementary information

Supplementary Information

## Data Availability

Data generated and analyzed during the current study are available from the corresponding author upon reasonable request.

## Code availability

The code for constructing the AHM Hamiltonian can be accessed via the link 10.5281/zenodo.17873350.
