# Supplementary material for: A hybrid Anyon-otto thermal machine
Source: npj Quantum Inf. 2026 Jul 24;12(1):117. doi: 10.1038/s41534-026-01328-6 (PMC13400308; doi:10.1038/s41534-026-01328-6)
Supplement: Supplementary file 1 — Supplementary Information [file 41534_2026_1328_MOESM1_ESM.pdf]

Supplementary Information: A Hybrid Anyon-Otto thermal machine

Mohit Lal Bera,[1,2] Joyce Kwan,[3] Armando Pérez,[1] Miguel A. García-March,[4] Ravindra Chhajlany,[5] Tobias Grass,[6,7] Maciej Lewenstein,[2,8] Utso Bhattacharya,[9] and Sourav Bhattacharjee[2,10,*]

[1]*Departamento de Física Teórica and IFIC, Universitat de València-CSIC, 46100 Burjassot (València), Spain*
[2]*ICFO–Institut de Ciéncies Fotóniques, The Barcelona Institute of Science and Technology, Av. Carl Friedrich Gauss 3, 08860 Castelldefels (Barcelona), Spain*
[3]*JILA, NIST, and Department of Physics, University of Colorado, Boulder, CO 80309, USA*
[4]*IUMPA - Instituto Universitario de Matemática Pura y Aplicada, Universitat Politècnica de València, E-46022 València, Spain*
[5]*Institute of Spintronics and Quantum Information, Faculty of Physics and Astronomy, Adam Mickiewicz University, 61614 Poznan, Poland*
[6]*DIPC - Donostia International Physics Center, Paseo Manuel de Lardizábal 4, 20018 San Sebastián, Spain*
[7]*IKERBASQUE, Basque Foundation for Science, Plaza Euskadi 5, 48009 Bilbao, Spain*
[8]*ICREA, Pg. Lluis Companys 23, 08010 Barcelona, Spain*
[9]*Institute for Theoretical Physics, ETH Zurich, Zurich, Switzerland*
[10]*Max Planck Institute for the Physics of Complex Systems, Nöthnitzer Str. 38, 01187 Dresden, Germany*

DERIVATION OF THE PERTURBATIVE GROUND STATE ENERGY E_{per} IN EQ. 9

Let us define $\{|C_\alpha\rangle\}$ as the basis of Fock states, where $|C_\alpha\rangle \equiv |n_{1,\alpha} n_{2,\alpha} \dots n_{j,\alpha} \dots n_{L,\alpha}\rangle$ and $\hat{n}_j |C_\alpha\rangle = n_{j,\alpha} |C_\alpha\rangle$. For $U \ll J$, the first order perturbative correction to the ground state energy is given by the expectation value of the interaction term in the ground state $|\psi_0\rangle$ (corresponding to $U = 0$),

$$\begin{aligned} E_{per} = \langle\psi_0|H_{int}|\psi_0\rangle &= \frac{U}{2}\sum_j \langle\psi_0|\hat{n}_j(\hat{n}_j-1)|\psi_0\rangle \\ &= \frac{U}{2}\sum_j\sum_{\alpha,\beta}\langle\psi_0|C_\alpha\rangle\langle C_\alpha|\hat{n}_j(\hat{n}_j-1)|C_\beta\rangle\langle C_\beta|\psi_0\rangle \\ &= \frac{U}{2}\sum_j\sum_\alpha |\langle C_\alpha|\psi_0\rangle|^2 n_{j,\alpha}(n_{j,\alpha}-1) \\ &= \frac{U}{2}\sum_j\sum_{n=0}^{N} n(n-1)\sum_\alpha |\langle C_\alpha|\psi_0\rangle|^2\delta_{n,n_{j,\alpha}} \\ &= \frac{U}{2}\sum_j\sum_{n=0}^{N} n(n-1)P_j(n), \end{aligned} \tag{S1}$$

where $P_j(n) = \sum_\alpha |\langle C_\alpha|\psi_0\rangle|^2\delta_{n,n_{j,\alpha}}$ is the probability of the jth site of the lattice being occupied with n particles in the ground state.

ADDITIONAL DETAILS ON THE HIGHER PROBABILITY OF MULTIPLE PARTICLE OCCUPANCY PER SITE AT $\theta \to 0, \pi$ FOR $N \gtrsim L/2$

For $\theta \neq 0$, the presence of the density-dependent phase in the hopping term (Eq. (1) of main text) implies a higher energy cost if multiple particles occupy the same site. For $N \gtrsim L/2$, the system cannot avoid such configurations resulting in a larger ground state energy except at $\theta \to 0, \pi$. To gain further intuition about the same, let us examine

* email: souravb@pks.mpg.de

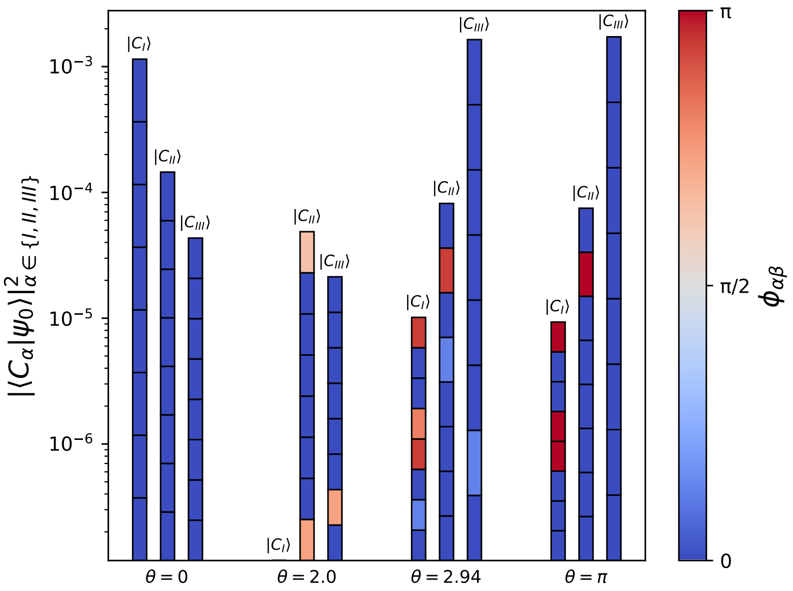

Figure S1. Probability of finding the ground state $|\psi_0\rangle$ in the Fock-space configurations $|C_{I,II,III}\rangle$ (see main text) at different values of θ. The blocks within each bar represent all the configurations in $\mathcal{S}_{\alpha=I,II,III}$, with the color of the blocks representing $\phi_{\alpha\beta}$. The numerical data for the plots are calculated for $L = 12$, $N = 6$, $J = 1.0$ and $U = 0$.

the probability of finding the ground state in a given Fock state $|C_\alpha\rangle$. Starting from the Schrödinger equation for the ground state, $H|\psi_0\rangle = E_0|\psi_0\rangle$, we find,

$$\begin{aligned}
|\langle C_\alpha|\psi_0\rangle|^2 &= \frac{1}{E_0^2}|\langle C_\alpha|H|\psi_0\rangle|^2 \\
&= \frac{1}{E_0^2}\Big|\sum_\beta \langle C_\alpha|H|C_\beta\rangle\langle C_\beta|\psi_0\rangle\Big|^2 \\
&= \frac{1}{E_0^2}\Big[\sum_\beta |\langle C_\alpha|H|C_\beta\rangle|^2|\langle C_\beta|\psi_0\rangle|^2 + \sum_{\substack{\beta,\gamma\\ \gamma\neq\beta}}\langle C_\alpha|H|C_\beta\rangle\langle C_\gamma|H|C_\alpha\rangle\langle C_\beta|\psi_0\rangle\langle\psi_0|C_\gamma\rangle\Big] \\
&= \frac{1}{E_0^2}\Big[\sum_\beta |\langle C_\alpha|H|C_\beta\rangle|^2|\langle C_\beta|\psi_0\rangle|^2 + \sum_{\substack{\beta,\gamma\\ \gamma<\beta}}\Big(\langle C_\alpha|H|C_\beta\rangle\langle C_\gamma|H|C_\alpha\rangle\langle C_\beta|\psi_0\rangle\langle\psi_0|C_\gamma\rangle + h.c.\Big)\Big] \\
&= \frac{1}{E_0^2}\Big[\sum_\beta |\langle C_\alpha|H|C_\beta\rangle|^2|\langle C_\beta|\psi_0\rangle|^2 + 2\mathrm{Re}\sum_{\substack{\beta,\gamma\\ \gamma<\beta}}\langle C_\alpha|H|C_\beta\rangle\langle C_\gamma|H|C_\alpha\rangle\langle C_\beta|\psi_0\rangle\langle\psi_0|C_\gamma\rangle\Big].
\end{aligned} \tag{S2}$$

In the non-interacting limit $U = 0$, we have $H = -J\sum_j(\hat{b}_j^\dagger e^{-i\hat{n}_j\theta}\hat{b}_{j+1} + \hat{b}_{j+1}^\dagger e^{i\hat{n}_j\theta}\hat{b}_j)$. For each $|C_\alpha\rangle$, we define S_α as the set of Fock-states such that,

$$\langle C_\alpha|H|C_\beta\rangle = \begin{cases} -J_{\alpha\beta}e^{-i\phi_{\alpha\beta}} & \text{if } |C_\beta\rangle \in S_\alpha \\ 0 & \text{if } |C_\beta\rangle \notin S_\alpha, \end{cases}$$

where the values of $J_{\alpha\beta}$ and $\phi_{\alpha\beta}$ depend on the direction of hopping of the particle in $\langle C_\alpha|H|C_\beta\rangle$. In general, for $|C_\alpha\rangle = |\ldots n_{j,\alpha}n_{j+1,\alpha}\ldots\rangle$ and $|C_\beta\rangle = |\ldots n_{j,\beta}n_{j+1,\beta}\ldots\rangle$, we have

$$J_{\alpha\beta} = J\sqrt{n_{j,\beta}(n_{j+1,\beta}+1)}, \quad \phi_{\alpha\beta} = -n_{j,\alpha}\theta \quad \text{for } n_{j,\beta}-n_{j,\alpha} = n_{j+1,\alpha}-n_{j+1,\beta} = 1 \quad \text{(right hopping)} \tag{S3}$$

$$J_{\alpha\beta} = J\sqrt{n_{j+1,\beta}(n_{j,\beta}+1)}, \quad \phi_{\alpha\beta} = n_{j,\beta}\theta \quad \text{for } n_{j,\beta}-n_{j,\alpha} = n_{j+1,\alpha}-n_{j+1,\beta} = -1 \quad \text{(left hopping)} \tag{S4}$$

Substituting in Eq. (S2), we have

$$|\langle C_\alpha|\psi_0\rangle|^2 = \frac{1}{E_0^2}\Big[\sum_{|C_\beta\rangle\in S_\alpha} J_{\alpha\beta}^2|\langle C_\beta|\psi_0\rangle|^2 + 2\mathrm{Re}\sum_{\substack{|C_\beta\rangle,|C_\gamma\rangle\in S_\alpha\\ \gamma<\beta}} J_{\alpha\beta}J_{\alpha\gamma}\langle C_\beta|\psi_0\rangle\langle\psi_0|C_\gamma\rangle e^{-i(\phi_{\alpha\beta}-\phi_{\alpha\gamma})}\Big]. \tag{S5}$$

The terms in the second summation in the above equation can destructively de-cohere for those $|C_\alpha\rangle$ which have high particle numbers per site, as for such states, $\phi_{\alpha\beta}-\phi_{\alpha\gamma}$ will fluctuate across the terms in the summation. It is therefore expected that such configurations will contribute to the ground state only if $\phi_{\alpha\beta}-\phi_{\alpha\gamma}=2m\pi$, for most of the configurations $|C_\beta\rangle, |C_\gamma\rangle \in \mathcal{S}_\alpha$, where m is an integer. For $\theta=0$, this is trivially satisfied and thus the probability for configurations with high occupancy per site is not reduced. However, in the limit $\theta\to\pi$, we have $\phi_{\alpha\beta}\to m\pi$ which allows for the existence of configurations for which $\phi_{\alpha\beta}-\phi_{\alpha\gamma}\to 2m\pi$. Thus, the probability of configurations with high occupancy per site is once again not prohibited in the limit $\theta\to\pi$, as in the case of $\theta=0$.

To illustrate the above, let us denote $\{|C_{\alpha,3}\rangle\}$ as the set of Fock-space configurations in which each of the configurations has at least one site with 3 particles. We examine the most-probable configurations $|C_{I,II,III}\rangle$ on this set, satisfying, $|\langle C_I|\psi_0\rangle|^2_{\theta=0} = \max|\langle C_{\alpha,3}|\psi_0\rangle|^2_{\theta=0}$, $|\langle C_{II}|\psi_0\rangle|^2_{\theta=2.0} = \max|\langle C_{\alpha,3}|\psi_0\rangle|^2_{\theta=2.0}$, and $|\langle C_{III}|\psi_0\rangle|^2_{\theta=\pi} = \max|\langle C_{\alpha,3}|\psi_0\rangle|^2_{\theta=\pi}$. Note that there can be more than one configuration satisfying the conditions above; however, the following discussion doesn't depend on which of those configurations is chosen. Figure shows the probabilities of finding the ground state in each of the above configurations at different values of θ. The blocks within each bar represent the configurations in $\mathcal{S}_{\alpha=I,II,III}$, with the color of the blocks representing $\phi_{\alpha\beta}$. For $\theta=2.0$, it can be seen that even for the most probable configuration $|C_{II}\rangle$, there exist configurations $|C_\beta\rangle$ in $\mathcal{S}_{\alpha=II}$ such $\phi_{\alpha\beta}\neq m\pi$. This results in a relatively smaller value of $|\langle C_{II}|\psi_0\rangle|^2$ as compared to the most probable configurations at $\theta=0,\pi$. As θ approaches the pseudo-fermionic limit (shown for $\theta=\pi-0.2$ and $\theta=\pi$ in Fig.), the probability of the configuration $|C_{III}\rangle$ grows in magnitude as $\phi_{\alpha\beta}\to 0,2\pi$ for all $|c_\beta\rangle$ in $\mathcal{S}_{\alpha=3}$.

ADDITIONAL DATA FOR WORK OUTPUT FOR DIFFERENT SYSTEM SIZES AND FILLING FRACTIONS AT LOW TEMPERATURE FOR $U\neq 0$

In this section, we provide additional data to show how the finite work output at low temperature in the weakly interacting limit ($0<U\ll J$) scales with system size. From Fig. S2, we can see that the optimum value of θ_1 for which the work is maximized does not appear to depend on the system size for a fixed filling. Furthermore, the maximum of the work is more pronounced for $N/L=2/3$ as compared to $N/L=1/2$.

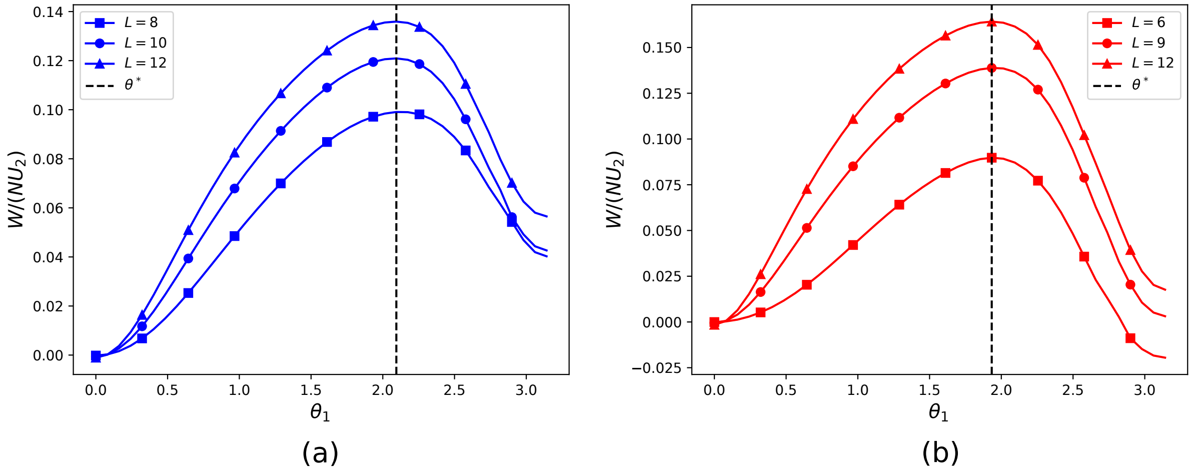

Figure S2. Low temperature ($T_A=T_B=0.1$) work output per particle (scaled with U_2) in the presence of finite interaction $U_2\neq 0$ as a function of θ_1 for different system sizes L and filling fraction (a) $N/L=1/2$ and (b) $N/L=2/3$. The parameters chosen for simulation are the same as those used in Fig. 4a of main text, i.e., $U_1=0$, $J=1.0$ and $\theta_2=0$.
